# Mitochondria as Potential Targets and Initiators of the Blue Light Hazard to the Retina

**DOI:** 10.1155/2019/6435364

**Published:** 2019-08-21

**Authors:** Jin-Xin Tao, Wen-Chuan Zhou, Xin-Gen Zhu

**Affiliations:** ^1^Department of Neurosurgery, The Second Affiliated Hospital of Nanchang University, Nanchang 330006, China; ^2^Department of Clinical Medicine, The Second Clinical Medical College, Nanchang University, Nanchang 330006, China

## Abstract

Commercially available white light-emitting diodes (LEDs) have an intense emission in the range of blue light, which has raised a range of public concerns about their potential risks as retinal hazards. Distinct from other visible light components, blue light is characterized by short wavelength, high energy, and strong penetration that can reach the retina with relatively little loss in damage potential. Mitochondria are abundant in retinal tissues, giving them relatively high access to blue light, and chromophores, which are enriched in the retina, have many mitochondria able to absorb blue light and induce photochemical effects. Therefore, excessive exposure of the retina to blue light tends to cause ROS accumulation and oxidative stress, which affect the structure and function of the retinal mitochondria and trigger mitochondria-involved death signaling pathways. In this review, we highlight the essential roles of mitochondria in blue light-induced photochemical damage and programmed cell death in the retina, indicate directions for future research and preventive targets in terms of the blue light hazard to the retina, and suggest applying LED devices in a rational way to prevent the blue light hazard.

## 1. The Retina and the Major Mitochondrial Distribution Pattern

**Figure 1:**
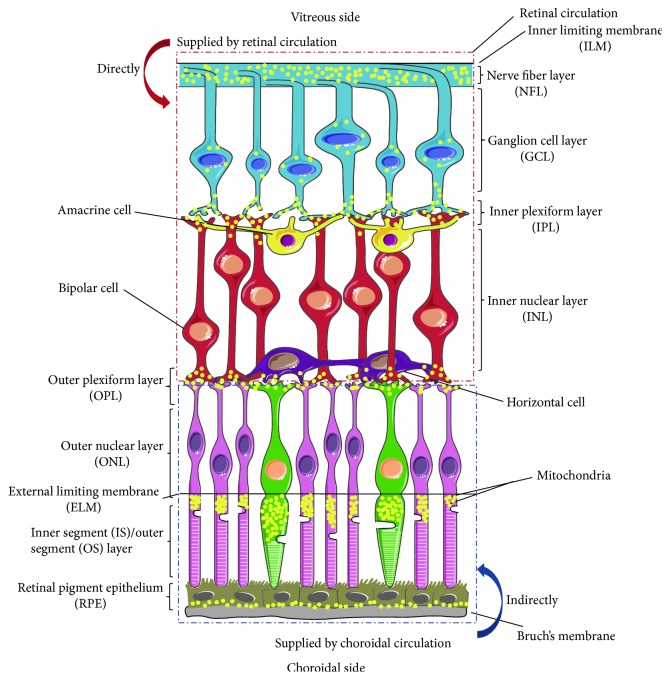
A schematic diagram of the retinal structure and a major distribution pattern of mitochondria. The human retina comprises ten distinct layers. The inner layers of the retina from the ILM to the dendrites of the horizontal and bipolar cells in the OPL are directly supplied with nutrients and oxygen through the retinal circulation, while the outer layers, from the RPE to the part of the OPL, as well as the fovea in primates, are indirectly supplied by the choroidal circulation across Bruch's membrane. Mitochondria are mainly located in the following retinal structure or cellular components: (1) NFL; (2) around RGC nuclei; (3) IPL and OPL, where numerous synapses are located; (4) the outermost portion of photoreceptor IS; and (5) the basal surface of RPE cells.

The retina is the innermost light-sensitive layer of the eye and is part of the central nervous system that originates as an outgrowth of the developing brain [1]. The neural retina is composed of several layers of neurons, including photoreceptor cells, bipolar cells, horizontal cells, amacrine cells, and ganglion cells, which are interconnected by synapses and responsible for the conversion of an image into electrical neural impulses transmitted to the cerebral cortex to form a visual perception. Similar to the brain, which consumes approximately 20% of the total oxygen used in the human body, the retina has tremendous oxygen demands for phototransduction and neurotransmission [2, 3]. To satisfy retinal energy requirements, two distinct vascular networks deliver the blood supply in the majority of mammals [4, 5]: (1) the retinal circulation consists of the vascular plexus that originates from the ophthalmic artery and is divided into three types, superficial, intermediate, and deep. The superficial plexus is at the nerve fiber layer (NFL), the intermediate plexus is near the proximal border of the inner nuclear layer (INL), and the deep plexus is proximal to the dendrites of the horizontal and bipolar cells in the OPL (outer plexiform layer) and distal to their somas. (2) The choroidal circulation indirectly supplies the outer layers of the retina (from the RPE to the part of the OPL, as well as the fovea in primates) via diffusion of nutrients and oxygen across Bruch's membrane. In particular, the OPL is served by both the inner retinal blood circulation and the choroidal flux. The choroidal blood supply to the avascular layers of the retina benefits from the rapid blood flow and high permeability of the choriocapillaris, which fuels most of the metabolism in the outer retina [6]. However, the choroidal circulation is not responsive to the metabolic state of retinal cells, rendering the retina avascular, especially the photoreceptor layer, which is vulnerable to both hypoxia and hyperoxia [7, 8]. As the ATP generated by oxidative metabolism in mitochondria is sufficient and required for neuronal survival and vitality, the abundant mitochondria associated with optimum functions are essential for the stability of the specialized neurons in the retina [9–11]. The retinal structure and the major mitochondrial distribution pattern are depicted in Figure 1.

Photoreceptor cells include cones and rods, which are responsible for photopic vision and scotopic vision, respectively. They share the same basic structure from the center to the inside/outside of the eye: (1) synaptic terminals, situated in the outer plexiform layer; (2) axons; (3) perikaryons; (4) inner segments; and (5) outer segments. The synaptic terminal of photoreceptors is specialized for intercellular signaling to the intermediate neurons, such as bipolar cells and horizontal cells. The K^+^/Na^+^ ATPases are located in the inner segment membrane and are required for the dark current in cells, serving as the basis for phototransduction. It was reported that, in humans, mitochondria are densely packed in the inner segments and are also found in axon terminals but are rare in photoreceptor somas [12]. Therefore, the distribution of mitochondria in photoreceptor cells is assumed to be associated with the demand for ATP. Indeed, the oxygen demands of photoreceptor cells are 3-4 times greater than those of other retinal and central nervous system neurons, and the estimates of the rate of oxidative metabolism in photoreceptor cells are among the highest in the human body. The cluster of mitochondria in the inner segment accounts for the intense oxidative metabolism of glucose there [13]. Stone and colleagues traced the migration of mitochondria during development and attributed mitochondrial polarization to oxygen-driven migration because they found that mitochondria can migrate only as far as the outer limiting membrane (OLM) before the inner segments develop; however, mitochondria pass through the OLM and locate as close as possible to the choriocapillaris after the inner segment forms [12]. They also proposed that the separation of the mitochondrial and nuclear genomes may be an important factor that makes photoreceptors more vulnerable to a range of genetic and environmental stresses; that is, the synthesis of the enzymes essential for the function of cells, such as for mtDNA repair and antioxidation, is highly dependent on the multifaceted interdependence of nuclear and mitochondrial genomes. This theory emphasizes the importance of mitochondria to the vitality of photoreceptor cells.

Retinal ganglion cells (RGCs) vary significantly in terms of their size, projections, and functions, but they share the following subcellular components in the retina: (1) the dendrites and synapses are located in the inner plexiform layers (IPL); (2) cell bodies are in the RGC layer; and (3) nonmyelinated axons are in the nerve fiber layer (NFL). In previous studies on the mitochondria in the human retina, high levels of mitochondria were found in the IPL and NFL and unequivocal labeling of mitochondria was observed in RGCs [14, 15]. Mitochondria also remain in the RGC layer where they localize around RGC nuclei [16]. Andrews et al. and Wang et al. further demonstrated that most mitochondria are concentrated within regularly spaced bulb-shaped areas of varicosity along the nonmyelinated RGC axon [15, 17]. Each human retina has approximately 96.6 million photoreceptors and approximately 0.7 to 1.5 million RGCs, and one RGC transmits the information from approximately 100 photoreceptors through its axon [18, 19]. This structure suggests that RGCs have high energy demands, which are satisfied by an abundance of mitochondria with optimum functions. Blocking the transported mitochondria at the lamellae of the lamina cribrosa may lead to the death of RGCs in glaucoma [20]. Mutations in Opa1, a mitochondrial fusion gene, contribute to synaptic loss observed in the IPL and optic atrophy [21]. These results suggest that the transport of mitochondria along the axons is critical to RGCs, from which long axons form the optic nerve, optic chiasm, and optic tract that connect to the brain.

Notably, in 2002, Berson and colleagues identified a new type of the photoreceptor from the subtypes of RGCs in mammals, which is known as the intrinsically photosensitive retinal ganglion cell (ipRGC). This type of cell accounts for only a very small subset of the ganglion cell population (less than 1% in primates) [22, 23]. Distinct from rods and cones, ipRGCs primarily transmit non-image-forming visual information and play a major role in the pupillary light reflex, release of melatonin, regulation of circadian rhythms, and other behaviors responsive to light [24–27]. These ipRGC functions depend on the presence of melanopsin, a light-sensitive pigment protein distributed throughout the cell membrane [28]. Most studies suggest that melanopsin has an absorption peak between 460 and 484 nm, which corresponds to blue light in the visible spectrum and forms the basis of the nonvisual biological effects of blue light [29]. In this way, retinal light exposure would increase if the pupillary reflex is understimulated by short-wavelength radiation, which would increase the risk of potential retinal damage [30].

The neural retina is supported by the outermost monolayer of retinal pigment epithelium (RPE) cells. Although RPE cells are polarized epithelial cells with no photosensitivity or neurotransmission function, they are able to maintain the structural integrity and visual function of the retina, particularly photoreceptor cells, through a system of efficient metabolic support and effective defense against photooxidative stress. The RPE constitutes a part of the blood/retina barrier with tight junctions between the lateral surfaces of epithelial cells [31]. This barrier contributes to immune privilege and enables highly selective transport between the blood and the subretinal space, which serves to supply nutrients, eliminate metabolites, and regulate ion and fluid homeostasis [32, 33]. Furthermore, RPE cells are involved in the regeneration of visual pigments in a process known as the visual cycle [34, 35]. In addition to metabolic support, RPE cells are also necessary to protect photoreceptors from photooxidative stress. RPE cells play a role in the phagocytosis of photoreceptor outer segments, which undergo constant destruction due to photooxidative damage [31, 36]. However, some undigested material is stored in lysosomes and accumulates with age in the form of a pigment called lipofuscin [37]. Additionally, melanosomes in the RPE can absorb scattered light and convert it into heat, which can be removed by the rapid choroidal blood flow [38]. Moreover, RPE cells contain high concentrations of nonenzymatic and enzymatic antioxidants. The mechanisms described above can diminish the photooxidative stress in photoreceptors to some extent. Stone et al. showed by electron microscopy that mitochondria congregate at the basal surface of RPE cells in the vascular areas of human retinas [12]. They proposed that the polarized distribution of mitochondria is a more reasonable and simple way to explain their proximity to the choriocapillaris since various ion channels are located in both the apical and basolateral membranes. In accordance with the vital importance of the RPE to the neural retina, especially the photoreceptor cells, mitochondrial dysfunction not only harms the RPE cells themselves but also affects adjacent photoreceptors. A similar mechanism is correlated with age-related macular degeneration (AMD) [39, 40].

## 2. LEDs and Potential Risks of Blue Light as a Hazard

Sunlight is composed of optical radiation that includes ultraviolet radiation (UVR, with a wavelength of 200-400 nm), visible radiation (visible light, with a wavelength of 400-760 nm), and infrared radiation (IR, with a wavelength of 760-10,000 nm) [41]. Blue light is generally defined as short-wavelength visible light ranging from 400 to 500 nm. Sometimes, blue light is further categorized as blue-violet light (approximately 400-440 nm) and blue-turquoise light (approximately 440-500 nm) [42]. Based on Planck's equation, the wavelength of light rays is negatively correlated with the amount of energy the rays contain, and hence, blue light has the highest energy in the visible light spectrum.

Photons are filtered sequentially when passing through each layer of intraocular structures until, finally, most of the light that reaches the retina is in the visible range (400-760 nm). The UVR below 295 nm is completely absorbed by the human cornea, and the UVR between 300 and 320 nm is very efficiently (nearly 100%) absorbed by both the cornea and the aqueous humor. Nearly one-half of the UVR between 320 and 360 nm is absorbed by both the cornea and the aqueous humor, and most of the other one-half is blocked by the lens. In addition to absorbing the near UVR (below 400 nm), the lens also absorbs far infrared radiation (above 800 nm). Near infrared radiation (NIR, 780-1400 nm) is mostly absorbed by the cornea [43]. IR in the range of 980-1430 nm is absorbed by both the cornea and the lens, and the portion above 1400 nm is absorbed by the vitreous humor [44]. Because of these advantageous absorption characteristics of the intraocular structures, the amount of potentially damaging UVR that reaches the retina is limited. However, the transmission of radiation at wavelengths longer than 400 nm increases rapidly. More than 65% of blue light at 460 nm is transmitted to the retina in children younger than 9 years old [43]. With age, modifications take place in lens proteins such that the crystalline lens becomes progressively less transparent and more deeply yellow, resulting in a relative reduction in the transmittance of visible light to the retina, especially in the blue region of the spectrum [45, 46]. This result was consistent with the findings of Artigas et al. [47]; they measured the spectral transmission of thirty-two excised human crystalline lenses in the age range of 41-77 years and one 30-year-old lens, and they found that the great variability in the spectral transmission of the human crystalline lens is greater for the lenses aged 60 and older, for which the transmission was 40% less for 420 nm wavelengths and 18% less for 580 nm wavelengths than it was for lenses between the ages of 40 and 59 years. Similarly, another study on the spectral transmission of human donor lenses in vitro reported that the transmission at 480 nm (corresponding to the peak absorption of melanopsin) was 82% in a 10-year-old lens and decreased to 56% in a 40-year-old lens and 23% in an 80-year-old lens. These results not only indicate the blue-light filtering properties of the aged crystalline lens, which is responsible for changes in sleep with age but also suggest a relative vulnerability of the younger retina to the blue light hazard [48].

Although sunlight is the major source of blue light, the advanced development of technological devices, especially light-emitting diodes (LEDs), with a relatively high level of blue light emission, is making blue light exposure increasingly common in modern environments. LEDs have been used widely for their inherent and potential advantages over previous technologies; in particular, they are smaller, have a longer lifespan and lower energy costs, and have faster switch and intelligent control functions. In addition to LED-based general illumination, LED-backlit liquid crystal displays (LCDs) (e.g., used for smart phones, computers, iPads, and e-readers) and LED displays (e.g., used for traffic signals and outdoor billboards), which are all dependent on LED technology, have been introduced to our lives in recent years. Therefore, LEDs are considered the most promising light sources and constitute the next generation of illumination [49]. The differences in the light emitted from the sun, LED-based devices, and artificial interior sources are discussed in depth in the article published by Behar-Cohen et al. [43]; the readers is referred to this article for more information.

LEDs generate white light mainly via the following two methods: (1) coating blue LEDs with yellow phosphors to form broad-spectrum white light that appears yellow when off and (2) mixing at least three LEDs that emit at different visible wavelengths, such as the red, green, and blue light of the spectrum, to produce white light [50, 51]. The majority of white LEDs available on the market are manufactured by the yellow phosphor method because this method is simpler and cheaper than the white light method. However, blue light from the yellow phosphor LEDs has a peak emission at approximately 450–470 nm, and the full width at half maximum is 30–40 nm, which is distinct from that of the daylight spectrum [52]. Additionally, the blue light emission from the white LEDs appears to increase with time, which corresponds to a rapid rise in the characteristic peak of blue light [53]. This may result from bleaching of the phosphors over time so that they absorb blue light less efficiently [54]. Moreover, the proportion of blue light increases with an increase in the color temperature of the LED backlight display [55]. Furthermore, the extensive use of LED-backlighted tablet displays has dramatically changed the way people read, since light directly illuminates the text in smart phones, computers, iPads, and e-readers rather than reflecting from the text on papers. These factors have recently increased the exposure of human eyes to blue light.

Previous studies in vivo have demonstrated that blue light from LEDs is associated with the retinal hazard. Shang et al. conducted experiments on Sprague-Dawley rats using exposure to full-spectrum white LEDs and blue LEDs (460 nm), and they found apoptotic and necrotic photoreceptors, as well as free radical production, which indicated that blue light induced serious photochemical injury of the retina [56]. Jaadane et al. used commercially available white LEDs and four different blue LEDs (507, 473, 467, and 449 nm) for exposure experiments on Wistar rats. They also found oxidative stress, retinal injury, and a loss of photoreceptors induced by LED light, and they concluded that the blue component of the LED was the major cause of the retinal damage [30]. Therefore, the extensive use of LED-based devices has raised great concerns among researchers, ophthalmologists, and the public about blue light hazards to the retina.

## 3. The Molecular Mechanisms of the Blue Light Hazard to the Retina

### 3.1. Photochemical Damage

The radiant energy contained in the form of photons can be absorbed by atoms or molecules only if the photon energy equals the energy demand for their excitation through transfer of the outermost electrons to higher orbital energy levels. The molecules with the ability to absorb radiation are dubbed chromophores. Activated chromophores dissipate the extra energy in various ways, for example, converting it to mechanical energy, heat, and chemical reactions, when they return to the ground state from their electronically excited state. Therefore, at least three mechanisms are involved in the light insult arising from radiation in the spectral range between 400 and 1400 nm: photomechanical, photothermal, and photochemical damages [57, 58].

Photomechanical damage is caused by high irradiance (megawatts to terawatts per cm^2^) and short exposure time (picoseconds to nanoseconds) and is not determined by the wavelength of light [59]. It is caused by the rapid energy absorption by the melanin granules in the RPE such that the heat does not dissipate and leads to the formation of lethal microcavitation bubbles in the cells [60, 61]. Such a mechanism is used therapeutically for iridotomy and capsulotomy with a YAG laser. The photothermal effect is also subject to irradiance thresholds and requires a relatively long exposure (microseconds to a few seconds) to reach thermal equilibrium [59]. Similarly, the original site of energy absorption is found in the melanin pigment that is located in the melanosomes of the RPE and the melanocytes of the choroids [62]. Such a mechanism is used, in part, in therapeutic laser photocoagulation (hemoglobin is also used) [63].

Photochemical damage occurs when the retina is exposed to incident radiation with a wavelength in the high-energy portion of the visible spectrum. Both acute and chronic exposures (months to years) can initiate photochemical damage because the photochemical effect relies on the total dose received, including the irradiance and the exposure duration [64]. Chromophores with the ability to produce chemical changes in another molecule are known as photosensitizers, and they can be excited by particular wavelengths of radiation and produce free radicals and ROS [65]. Radiant energy absorbed by photosensitizers can be subsequently used to break bonds in other molecules via direct electron exchange or direct hydrogen exchange, which leads to the formation of free radicals [66, 67]. Energy transfer from the excited state of a photosensitizer to oxygen generates singlet oxygen (^1^O_2_), while electron transfer from a photoexcited photosensitizer and subsequent reactions generate superoxide radicals (O_2_^•-^), hydrogen peroxide (H_2_O_2_), and hydroxyl radicals (^•^OH). These described molecules, derived from oxygen, are known as reactive oxygen species (ROS) [68]. ROS are lethal for cells due to the following main effects [43]: (1) lipid peroxidation, a process in which membranous structures are catabolized through an attack on the polyunsaturated fatty acids in a lipid such that the retina is severely damaged due to a large accumulation of cell membranes [69]; (2) genetic mutations; and (3) inactivation of enzymes and proteins, which is a mechanism used in photodynamic therapy (PDT) [70]. Blue light-induced retinal damage, also known as retinal phototoxicity or blue light hazard, is commonly due to photochemical damage [41]. These findings on ROS are consistent with those from many published studies suggesting that blue light induces a significant increase in ROS production in the retina and that the blue light hazard to the retina can be diminished by antioxidants [57, 71, 72].

### 3.2. Chromophores in the Retina Facilitate Photochemical Damages

As previously described, the retina is characterized by (1) high oxygen consumption, (2) exposure to a large dose of radiation, (3) a large concentration of polyunsaturated fatty acids, which are particularly abundant in the cell membrane and are among the targets of ROS. These factors dramatically increase retinal susceptibility to photochemical damage. Moreover, numerous endogenous chromophores in the retina also constitute a major factor responsible for photochemical damage [66]. We have summarized the major chromophores with peak wavelength absorption close to that of blue light (400-500 nm) in the retina and the mitochondria, as demonstrated in Table 1. As shown in Table 1, the majority of chromophores contribute to the blue light hazard.

Studies in vitro showed that all-*trans*-retinal that is involved in the visual cycle and lipofuscin that accumulates in aged RPE cells plays roles in retinal photochemical damage [73, 74]. It was observed that the degree of the blue light hazard to the retina is positively correlated with the rhodopsin content in the photoreceptors [75]. It has been suggested that rhodopsin mediates photoreceptor damage, since both photoreversal of rhodopsin bleaching and production of toxic rhodopsin intermediates increased after exposure to blue light (403 ± 10 nm) [76]. However, blue light has no impact on the retina when rhodopsin regeneration is blocked by the depletion of the protein RPE65 [77]. As visual pigments, both rhodopsin and cone opsin are composed of a protein component and a bound chromophore, 11-*cis*-retinal, which is photoisomerized to all-*trans*-retinal (atRal) by the light reaching the retina. All-*trans*-retinal is highly reactive in its free form, and it acts as an efficient photosensitizer to produce ROS and free radicals when exposed to short-wavelength visible light in studies in vitro [78]. Moreover, the activation of retinal opsins such as melanopsin (OPN4) by blue light can also generate ROS via the phospholipase C pathway, in a process that is partly responsible for the blue light hazard [79–81]. Lipofuscin-induced phototoxicity was shown to be wavelength-dependent, at least in vitro. The lipofuscin granules isolated and purified from human RPE (hRPE) cells generated superoxide anions most effectively upon exposure to the spectrum of blue light (400-520 nm) [82]. Furthermore, exposure of the lipofuscin-loaded hRPE cells to blue light (400-550 nm) caused a series of alterations, such as lipid peroxidation and protein oxidation, that culminated in cell death, while light exposure of these cells at 550 to 800 nm had no adverse effect [83]. For more information on the photoreactivity and spectral dependence of RPE lipofuscin, please refer to review article from Boulton et al. [84]. A_2_E, the first isolated and identified component from RPE lipofuscin, absorbs the light mostly in the blue light range and can act as a photosensitizer of ocular lipofuscin with the ability to trigger ROS production and induce cell apoptosis [85–88]. However, the extent of the A_2_E contribution to lipofuscin photoreactivity has been questioned [89]. It was reported that the spatial distribution of A_2_E and the lipofuscin fluorescence in the hRPE were not correlated because strong RPE fluorescence was observed in the central area of the hRPE, while the concentration of A_2_E was greatest in the far periphery and decreased toward the central region [90]. A comparative experiment conducted by Shamsi and Boulton further demonstrated that lipofuscin granules were at least 2 orders of magnitude more photoreactive than the endogenous A_2_E equivalent, confirming the weak direct photoreactivity of A_2_E [91]. Since the phototoxic properties of A_2_E in hRPE cells were less active than those of all-*trans*-retinal, which is the precursor of A_2_E, the endogenous production of A_2_E was even proposed to be a protective mechanism for detoxifying the cells from the more reactive free all-*trans*-retinal [92].

Fortunately, the retina is also equipped with pigments that are likely to prevent eyes from the blue light hazard. According to studies in vitro, lutein and zeaxanthin, located in the peripheral retina and the macula, respectively, may protect the retina from the blue light hazard mainly by (1) preventing A_2_E oxidation and ROS production, acting as ROS scavengers, and significantly reducing lipofuscin formation in RPE cells [93, 94] and (2) enhancing the activity of other antioxidants, in contrast to all-*trans*-retinal and lipofuscin, which cause a dramatic decrease in the potential of the antioxidant system [95].

However, the role of melanin in the melanosomes in RPE cells is ambiguous. On the one hand, it is believed that melanin exhibits a protective effect via absorption of scattered light, regulation of antioxidant enzymes, and antioxidative properties [96–101]. On the other hand, substantial evidence suggests that melanin induces the phototoxicity of RPE cells, especially in aged cells, leading to increased production of various types of ROS and free radicals [102–106]. Sarangarajan and Apte proposed that the propensity of melanin to act as an antioxidant is proportional to its degree of polymerization or molecular weight and the number of prooxidative melanin polymers increases with age, but more investigations are required to definitively support or refute such a hypothesis [107].

In addition, phototoxicity is conferred by the culture medium, especially Dulbecco's modified Eagle's medium (DMEM), which is widely applied to the culture of retinal cells in vitro and should be excluded as a factor responsible for the damaging effects of light. It was reported that the outgrowth of neurites from retinal explants was inhibited when culture medium containing 1 *μ*M riboflavin was exposed to ultraviolet light (UV-C, 30 W, Phillips, effective radiated power of 740 mW/cm) for 12 h [108]. A lethal effect was observed when unirradiated mammalian cells were incubated in DMEM previously irradiated by near-ultraviolet (peaking at 365 nm, exposure intensity of 4 *μ*W/mm^2^), and this effect was attributed to riboflavin and tryptophan or riboflavin and tyrosine in the DMEM [109]. Therefore, it is necessary for future researchers to pay attention to potential medium-mediated phototoxic effects due to the photosensitizers possibly present in the culture medium.

### 3.3. Chromophores in the Retina Potentially Affect Mitochondria

Numerous studies have shown that chromophores in the retina have a negative impact on the structure and function of mitochondria. Siems et al. incubated mitochondria that were isolated from a rat liver with five types of beta-carotene cleavage products, including retinal and mixtures with retinal. They found that retinal targets mitochondria at three points: (1) the respiratory chain, where 1 *μ*M retinal inhibited oxidative phosphorylation by 12.4 ± 0.5%, and retinal at concentrations as low as 0.5 *μ*M inhibited phosphorylation by 6.3 ± 2.9%; (2) the antioxidants in mitochondria, for which retinal or retinal cleavage products induced extensive decreases in glutathione (GSH), marked losses of total protein-SH, and a threefold increase in the GSSG/GSH ratio (GSSG is an oxidized form of GSH) compared to the respective controls; and (3) mitochondrial membranes, where malonic dialdehyde (MDA) formation was enhanced, implying lipid peroxidation of mitochondrial membranes [110]. Moreover, bisretinoid at a concentration less than 5 *μ*M impaired the function of isolated cytochrome oxidase [111]. All-*trans*-retinal at a concentration greater than 50 *μ*M was observed to uncouple oxidative phosphorylation in mitochondria isolated from rat hearts [74]. Compared with that induced by retinal and retinoid, A_2_E-induced toxicity in RPE mitochondria led to contradictory results. A fragmented mitochondrial network was observed in 25 *μ*M A_2_E-laden ARPE-19 cells, which is comparable to that present in RPE cells from human eyes [112]. Another study performed on human RPE-J cells revealed that the incubation of 50 *μ*M A_2_E for 6 h, an amount that mimics the A_2_E load of RPE lysosomes found in healthy elderly people, could diminish ATP synthesis in mitochondria [113]. It was reported that A_2_E at levels greater than 10 *μ*M inhibited respiration of the mitochondria isolated from a rat liver, and this inhibition could be overcome by adding cytochrome c or cardiolipin, which suggests that A_2_E prevents cytochrome c binding to the inner mitochondrial membrane and thus interrupts the electron flow between cytochrome bc1 and COX [86]. However, it was also reported that A_2_E did not affect the metabolic functions in mitochondria isolated from rat hearts, even at a concentration as high as 600 *μ*M, while all-*trans*-retinal at doses greater than 50 *μ*M both inhibited mitochondrial oxidation and uncoupled oxidative phosphorylation, suggesting that A_2_E production may suppress the toxicity-induced all-*trans*-retinal [74]. Among the factors contributing to the different findings, distinct oxidizable substrates and different measurements were used in the experiments. The former study measured oxygen consumption by mitochondria respiring succinate, while the latter study used glutamate as the oxidizable substrate, and the following oxidative properties of mitochondria were measured: state 3/state 4 respiratory rates, respiratory control ratios (state 3/state 4), ADP to atomic oxygen phosphorylation ratio, and dinitrophenol-induced oxidation. In addition, it was proposed that opsins such as melanopsin activated by blue light at 420 nm could open TRP (transient receptor potential) calcium ion channels in the cell membrane, leading to calcium influx and ROS production in mitochondria [114].

## 4. The Roles of the Mitochondrial Respiratory Chain in the Blue Light Hazard

### 4.1. A Delicate Balance between the Oxidant and Antioxidant Systems

The mitochondrial respiratory chain is a major intracellular source of ROS generation, accounting for almost 90% of the total ROS found under normal physiological conditions in mammalian cells, and only a small fraction of these ROS originate from NADPH oxidase, 5-lipoxygenase, and enzymes that react with oxygen as the substrate [115–117]. Electrons that leak from the respiratory chain interact with O_2_ directly, which leads to the production of superoxide anion (O_2_^•-^) when O_2_ is partly reduced by receiving a single electron [118, 119]. These ROS produced from the respiratory chain can be released into the cytoplasm or the mitochondrial matrix in which mitochondrial DNA and a variety of enzymes involved in nutrient metabolism affect mitochondrial and cellular functions. Fortunately, a well-developed antioxidant defense system composed of enzymatic and nonenzymatic antioxidants would protect cells against oxidative stress via elimination of ROS [120–122]. Enzymatic antioxidants, including superoxide dismutase (SOD), catalase, glutathione reductase (GR), and glutathione peroxidase (GPx), are primarily distributed in the cytoplasm, mitochondria, and other organelles, such as peroxisomes and microsomes [123, 124]. Nonenzymatic antioxidants mainly comprise vitamin C, vitamin E, *β*-carotene, glutathione (GSH), and other small molecule ROS scavengers [123, 125]. The enzymatic and nonenzymatic systems synergistically account for the major antioxidant effects in cells and maintain a delicate balance with ROS generation [121, 122]. Marie et al. showed that blue light exposure (415-455 nm, 15 W/(m^−2^ sr^−1^)) for 15 h led to a decrease in SOD and catalase activities and an increase in GSSG and ROS in A_2_E-loaded RPE cells [126], suggesting that the delicate balance between oxidant and antioxidant systems is easily disrupted by a large dose of blue light radiation.

### 4.2. Chromophores in the Mitochondrial Respiratory Chain Facilitate the Blue Light Hazard

**Figure 2:**
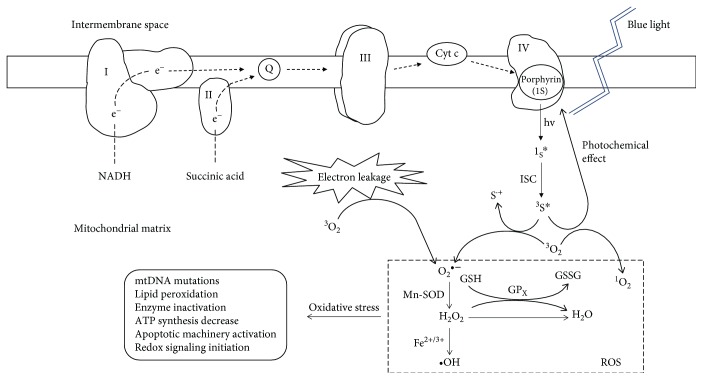
Possible mechanisms of blue light-induced ROS generation in mitochondria. On the one hand, absorption of blue light by the endogenous chromophores in the respiratory chain excites the chromophore (for example, porphyrin) from its ground state (^1^S) to a transient singlet state (^1^S∗) and then to the triplet state (^3^S∗) via intersystem crossing (ISC). Porphyrin in the triplet state can transfer an electron to an oxygen molecule present in the mitochondria; as a result, the oxygen molecule is activated from its ground state (^3^O_2_) to become a superoxide anion (O_2_^•-^), and a photosensitizer radical cation (S^•+^) is produced at the same time. In addition, the excited triplet state of porphyrin (^3^S∗) can return to its ground state (^1^S) by energy transfer to an oxygen molecule (^3^O_2_), which leads to the generation of singlet oxygen (^1^O_2_). On the other hand, blue light disrupts electron transport and increases the leakage of electrons; these electrons interact with O_2_, which leads to the production of O_2_^•-^ as well. The O_2_^•-^ produced by either method can be subsequently converted into other forms of ROS by receiving more electrons in the mitochondria. In addition, O_2_^•-^ and H_2_O_2_ can be, respectively, detoxified by Mn-SOD and GPx, the major antioxidases in the mitochondria. However, the hydroxyl radical (^•^OH) cannot be eliminated through an enzymatic reaction, even though, among the ROS, it is characterized as having the greatest toxic effect on the tissue; it has a very short half-life in vivo. A wide range of mitochondrial oxidative damage is realized when the antioxidant system cannot counteract ROS generation.

It was reported that isolated mitochondria exposed to blue light generated singlet oxygen, superoxide anions, and hydroxyl radicals, indicating the photosensitizing properties of whole mitochondria in vitro [127]. As summarized in Table 1, respiratory complexes located in the inner mitochondrial membrane harbor two major chromophores, flavin and porphyrin, both of which have an absorption peak in the blue light range. Light-activated flavins can induce the oxidation of several substances, such as amino acids and glucose, and mediate H_2_O_2_ production, which leads to lipid peroxidation and protein crosslinking [128–130]. The flavin is often attached to other groups and is present in the forms of FAD and FMN (a phosphorylated riboflavin). Many flavin-containing proteins called flavoproteins have either FMN or FAD as a prosthetic group, and they mainly perform redox reactions in the mitochondria [131, 132]. The common flavoproteins, respiratory complex I and complex II, have been reported to be the underlying mechanisms for the H_2_O_2_ production induced by blue light with an absorption peak of approximately 450-520 nm [130]. Aggarwal et al. found that light in the range of 400-500 nm was most effective O_2_-dependent mitochondrial inactivation, which was greatly enhanced by the presence of free flavins, suggesting that visible light mediated a flavin-photosensitized reaction [133]. Porphyrins are often applied to photodynamic therapy as effective photosensitizers, since porphyrin triplets activated by the photons at the wavelengths of blue-violet light can interact with molecular triplet oxygen (^3^O_2_) to form radicals and ROS and acutely injure cellular constituents, including unsaturated lipids, amino acid residues, and nucleic acids, causing targeted cell death [134–136]. The scheme of photosensitizing reactions in mitochondria is shown in Figure 2. Many porphyrin-containing proteins have heme as a prosthetic group such that they are known as hemoproteins and include hemoglobin, catalases, and cytochromes [137]. Complex III contains cytochrome b (Cyt b) and cytochrome c_1_ (55 c_1_). In addition, cytochrome P450 located in the inner mitochondrial membrane has a heme iron center [138]. Complex IV, also named cytochrome oxidase (COX) or cytochrome c oxidase (CCO), has a Cyt a and a Cyt a_3_ and an absorption peak at approximately 420 nm in the oxidized form and at approximately 440 nm in its reduced form [139].

Numerous studies have indicated that the electron transport chain may act as one of the targets of blue light. The expression of COX in the rat retina was irreversibly inhibited after exposure to spectral blue light at 404 nm and led to retinal damage [140]. COX is expected to function as a predictor of retinal metabolism after blue light exposure in vivo since the COX activity was found to be significantly altered in different layers of the SD rat retina over time, while no visible retinal morphological changes were observed by ophthalmoscopy or light microscopy [130, 141]. Nunez-Alvarez et al. treated ARPE-19 cells with well-defined toxins with four mitochondrial enzyme complexes and assessed cell death to determine whether blue light affects these mitochondrial enzyme complexes specifically; they eventually concluded that at least two out of the four respiratory complexes are involved in the blue light hazard [142]. del Olmo-Aguado et al. proposed that direct action of blue light on mitochondrial respiration causes ATP depletion, whereas indirect action contributes to oxidative stress [143]. The blue light hazard was attenuated in fibroblasts when their mitochondrial respiratory chain function was perturbed by incubating them for 40 days in culture that contained ethidium bromide [10]. Calzia et al. irradiated whole mouse eyeball cultures with blue light and found that ATP synthesis, electron transfer capacity, and the activity of complex I and complex II were impaired, while ROS production increased in the purified rod outer segment (OS) [144]. It was reported that the blue light phototoxicity, manifested as the breakdown of the blood-retina barrier in albino rabbits, was likely mediated by cytochrome oxidase in the RPE [145].

### 4.3. Mitochondrial DNA Lesions Affect the Respiratory Chain in Turn

The vulnerability of mitochondrial DNA (mtDNA) is also associated with damage to the mitochondrial respiratory chain. It is generally believed that the mtDNA damage is more extensive and more persistent than the nuclear DNA damage, mainly because of the following characteristics of the mtDNA genome: (1) close proximity to a high steady-state level of ROS in the mitochondrial matrix, (2) lack of histones and introns, and (3) a high transcription rate but less efficient mtDNA repair mechanisms [146]. The suggestion of a greater mtDNA susceptibility is supported by the Godley et al. study, which showed maximum mtDNA damage in human primary retinal epithelial (hRPE) cells exposed to visible light (390-550 nm, 2.8 milliwatts/cm^2^) for 3 h but no nuclear DNA lesions in cells cultured under the same conditions [127]. Many studies have demonstrated that, in addition to increased ROS generation, significant mtDNA damage was observed in blue light-exposed retinal cells [127, 147–149]. Moreover, the superoxide anion (O_2_^•-^) was confirmed to be the primary species that led to the mtDNA lesions [127], which suggests that respiratory chain-derived ROS can mediate mtDNA damage. Unfortunately, oxidative stress-mediated damage to mtDNA can in turn lead to the accumulation of mutations that affect the integrity of the respiratory chain, since mtDNA primarily encodes 13 structural proteins that are components of the respiratory chain [150, 151].

In summary, blue light can potentially affect the mitochondrial respiratory chain and dramatically increase ROS generation, likely throughout one of two methods (Figure 2): (1) photochemical effects mediated by local photosensitizers or (2) an increase in electron leakage caused by damage to the electron transport chain. Mitochondria are distributed extensively in the retina and are exposed directly to blue light. As the major intracellular source of ROS, the mitochondrial respiratory chain has peak absorption in the spectrum of blue light, which increases ROS generation and the possibility of subsequently developing oxidative stress. Therefore, the mitochondrial respiratory chain appears to be one of the potential targets and initiators for blue light-induced oxidative stress in the retina.

## 5. The Roles of Mitochondria-Involved Cell Death in the Blue Light Hazard

It is generally recognized that blue light can lead to photochemical damage and ultimately trigger cell death in a diverse range of cell types. Based on this knowledge, photodynamic therapy (PDT), which includes an addition of an exogenous photosensitizer and subsequent blue light irradiation, is approved as an evolving therapeutic modality for some defined diseases such as cancers [70, 101, 134]. Instead, in the field of ophthalmology, more attention has been concentrated on the prevention of the blue light hazard; thus, it is necessary to reveal the underlying mechanisms involved in blue light-induced retinal damage. A previous study has demonstrated that blue light-mediated retinal cell death is associated with the generation of ROS and their negative effects on lipids, nucleic acids, and proteins [152]. Furthermore, H_2_O_2_ and blue light were found to cause significant oxidative damage and cell death via a similar molecular mechanism in ARPE-19 cells [153]. Recently, Nunez-Alvarez and Osborne reported that the underlying mechanism of blue light negatively affecting R28 cell survival involves loss of mitochondrial status and oxidative stress [154]. Therefore, oxidative stress secondary to the photochemical effects is thought to be one of the most important cytotoxicity mechanisms underlying blue light-induced retinal cell death [43]. To date, studies have proposed two predominant forms of programmed cell death, apoptosis and necroptosis, to explain blue light-induced cell death in the retina. However, it remains uncertain which factors determine the propensity of cell death by either apoptosis or necroptosis. It was proposed that blue light only affects cells with intact mitochondrial respiratory chain function, suggesting that mitochondria are required for light insult [10, 155]. Indeed, mitochondria are regarded as the junctions of multiple cell death pathways in mammalian cells [156].

### 5.1. Mitochondria Participate in Blue Light-Induced Apoptosis

**Figure 3:**
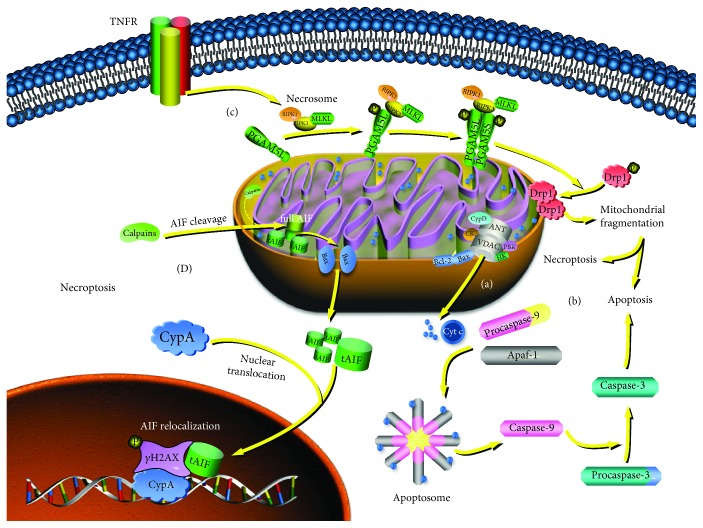
Possible pathways of cell death that involve the mitochondria in the presence of oxidative stress. (a) Molecular composition of the mPTP complex. A classic model of the mPTP is illustrated in this figure. mPTP spans the mitochondrial inner and outer membrane, and its core components are formed by ANT and VDAC. Several molecules are mainly involved in the regulation of mPTP: (1) CypD, located in the mitochondrial matrix; (2) creatine kinase (CK), an intermembrane space protein; (3) the outer mitochondrial membrane proteins, including HK and PBR, and those of the Bcl-2 family, such as Bax and Bcl-2. (b) Mitochondria-associated intrinsic apoptosis pathway. The opening of mPTP causes mitochondrial permeabilization; thus, proapoptotic proteins located in the mitochondrial intermembrane space, such as Cyt c, are released into the cytoplasm. Cyt c binds to Apaf-1 to form the apoptosome, which is responsible for the recruitment and cleavage of procaspase-9 to active caspase-9. Subsequently, caspase-9 triggers the downstream caspase cascade, resulting in apoptosis. (c) Mitochondria-associated intrinsic necrosis pathway. The necrosome is formed by RIPK1, RIPK3, and MLKL in the extrinsic necrosis pathway and binds sequentially to PGAM5L and PGAM5S. PGAM5S directly recruits and activates Drp1 on mitochondria, contributing to mitochondrial fragmentation, a common step for both apoptosis and necrosis. (d) AIF-mediated necroptosis pathway. Mitochondrial calpains or cytoplasmic calpains that are translocated to mitochondria in an unclear manner can cleave the full AIF that is embedded into the inner membrane to soluble tAIF, which is transferred across the outer membrane through the Bax protein poles. Chaperoned by CypA, tAIF is redistributed to the nuclear compartment, leading to chromatinolysis, which is regulated by *γ*H2AX and cyclophilin A (CypA).

Oxidative stress has been reported to cause excess retinal cell loss and mediate the initiation of apoptosis in cultures in vitro and animal models [157–160]. There are two major pathways involved in apoptosis: the extrinsic pathway mediated by cell membrane receptors and the mitochondria-associated intrinsic pathway [10]. The intrinsic mitochondrial pathway may act as the key regulatory mechanism in oxidative stress-triggered apoptosis, which is associated with the status of the mitochondrial permeability transition pore (mPTP) [147, 161]. mPTP is a proteinaceous pore complex present at the translocation contact site between the mitochondrial inner and outer membranes [162] (Figure 3(a)), and its abnormal opening triggered by proapoptotic signals can cause mitochondrial permeabilization. Consequently, a large number of apoptosis-related proteins are released from the mitochondrial intermembrane space or inner membrane into the cytoplasm. These proteins either activate specific caspases that initiate downstream apoptotic cascades (such as Cyt c, AIF, Smac/Diablo, and procaspases) or destroy nuclear chromosomes in a caspase-independent manner (such as Omi/HtrA_2_, AIF, and EndoG), leading to irreversible apoptosis [156, 163]. The caspase family can be roughly divided into two subgroups according to their involvement in the different phases of the apoptotic signaling pathway. One caspase subfamily acts as an upstream initial signal and includes caspase-2, -8, -9, and -10; the other caspase subfamily consists of caspase-3, -6, and -7, which participate in downstream executive signal pathways [164]. Caspase-8 is able to cleave Bid to tBid, which connects the extrinsic apoptotic pathway with the intrinsic pathway [165]. It is notable that caspase-9 is the typical caspase upstream of the intrinsic apoptotic pathway. Cytochrome c released from mitochondria can bind to apoptotic protease activating factor-1 protein (Apaf-1) in the cytoplasm to form the apoptosome, which subsequently recruits and cleaves procaspase-9 to caspase-9, leading to the activation of downstream executive caspases [163] (Figure 3(b)). The Bcl-2 family plays an important role in the regulation of the mitochondria-dependent apoptotic program. In general, Bcl proteins are located in the mitochondrial outer membrane, and the Bcl-2 set of proteins comprises three subgroups based on their distinct properties. The Bax subfamily (e.g., Bax and Bak) and Bcl-2 homology 3 (BH_3_) subfamily (e.g., Bad and Bid) serve as proapoptotic components, while the Bcl-2 subfamily (e.g., Bcl-2, Bcl-xL, and Mcl-1) is known for its antiapoptotic role. These proteins maintain a delicate balance under normal physiological conditions and, when triggered by apoptotic stimuli such as ROS products, mediate mitochondrial outer membrane permeabilization and apoptotic susceptibility by inducing the mPTP opening or directly forming ion transport channels across the mitochondrial membrane [166–168].

Among all studies on the blue light hazard to the retina, the most frequently adopted experimental model comprises RPE cells, in which apoptosis is believed to be involved in geographic atrophy AMD (age-related macular degeneration) [169]. Most of these studies have revealed ROS accumulation and the proteolytic caspase cascade in the RPE cell death program; specifically, (cleaved) caspase-9 and (cleaved) caspase-3 proteins have been shown to increase under blue light compared to those that were treated in the dark, suggesting that caspase-dependent apoptosis occurs in blue light-induced RPE cell death [152, 153, 170, 171]. Furthermore, blue light is observed to increase the release of cytochrome c and attenuate the ratio of Bcl-2/Bax, which represents the ability of cells to inhibit apoptosis and block the negative effects of ROS on mitochondria, indicating that mitochondria play a critical role in RPE apoptosis [152, 172]. This finding is similar to that reported for the case of RGC-5 exposed to long-term blue light in which active caspase-3 (p17) and ROS-sensitive proteins (Nrf2 and HO-1) were elevated, while the ratio of Bcl-2/Bax was decreased [173]. However, it has been proposed that, in some cases, oxidative stress-induced apoptosis of RGCs is caspase-independent because the activated caspase was not detected and the addition of Z-VAD-fmk (a caspase inhibitor) had no effect. However, the mitochondrial uncoupler M3778 significantly reduced light-induced apoptosis, which implies that the mitochondrial respiratory chain is required for light-induced caspase-independent apoptosis [174]. Indeed, in addition to ATP generation, oxidative phosphorylation is necessary for the formation of the apoptosome in the apoptosis signaling pathway [175]. It is difficult to culture photoreceptor cells in vitro due to their high susceptibility to phototoxic effects, which restricts further study on them to some extent, but apoptosis was also observed via TUNEL staining, immunohistochemistry, and transmission electron microscopy as the major mechanism involved in photoreceptor demise in a blue light-irradiated rat model [56, 161].

A loss of mitochondrial membrane potential (MMP) is found in many apoptotic systems and may be an early event in the apoptotic cascade [176–178]. Mitochondrial function is highly dependent on the MMP [126]. The maintenance of MMP relies on electrochemical potential, which is generated when respiratory complexes I, III, and IV pump protons out of the mitochondrial matrix across the inner mitochondrial membrane in a process accompanied by ATP synthesis [179, 180]. As a consequence, decreased MMP (termed mitochondrial membrane depolarization) refers to a decrease in energy metabolism in mitochondria, while increased MMP (termed mitochondrial membrane hyperpolarization) refers to an increase in electron transport and will produce more ROS as byproducts [114, 181]. A decrease in MMP seems common in blue light-induced retinal photochemical damage. Nunez-Alvarez et al. demonstrated a loss of cell viability, production of ROS, and mitochondrial depolarization in ARPE-19 cells exposed to blue light of 465-475 nm [142]. Roehlecke et al. studied the effects of sublethal doses of blue light irradiation on human RPE (hRPE) cells and found elevated intracellular ROS levels, overexpressed stress-related proteins, and significantly reduced MMP [179]. Li et al. also found decreased MMP and increased ROS in retinal neuronal (R28) cells exposed to blue light of 450 nm [182]. The studies described above showed that decreased MMP appears to be associated with increased ROS in the blue light-exposed retina. Indeed, increased ROS may reach a threshold level that triggers the abnormal opening of mPTP by upregulating Bax expression and thus leads to a simultaneous depletion of MMP [156, 183]. In addition, activated mPTP can cause the release of Cyt c and AIF, a finding that is consistent with the classic view that apoptosis is irreversible once the MMP is depleted [177]. Recently, an interesting study revealed that blue light triggered a slow translocation of intracellular calcium into the mitochondria, followed by a transient change in mitochondrial membrane hyperpolarization and subsequent mitochondrial membrane depolarization, which indicates that blue light may affect MMP via calcium influx [176]. Moreover, Argun et al. found that blue light-induced ARPE-19 apoptosis was clearly associated with increased levels of intracellular Ca^2+^ [184]. It is assumed that the concentration of free calcium ions in mitochondria determines the status of the mPTP (open or closed), and calcium overload stimulates the opening of mPTP, leading to decreased MMP and caspase-dependent apoptosis [176]. In general, as proposed by Ly et al., it seems that the loss of MMP may be an early requirement rather than a consequence of apoptosis [185].

### 5.2. Mitochondria Participate in Blue Light-Induced Necroptosis

Even though apoptosis is the preferred form of cell death and the best-known mechanism involved in retinal degeneration [30], there are also a few studies indicating that necroptosis plays a major role in oxidative stress-triggered retinal cell death. Li et al. treated ARPE-19 with high concentrations of H_2_O_2_ and, on the basis of cell morphology, described cell death as programmed necrosis [186]. Similarly, Hanus et al. found that necrostatins, which interact with receptor-interacting protein kinase (RIPK) inhibitors, could prevent the RPE cell death triggered by oxidative stress, but z-VAD, a caspase inhibitor, cannot suppress necrosis, indicating that the nature of RPE cell death is necroptosis [187].

Necroptosis is also described as programmed necrosis, regulated by RIPK and manifested with morphological features of necrosis, including cell and organelle swelling, plasma membrane rupture, and necrosome formation, while apoptosis is characterized by cell shrinkage, nuclear fragmentation, loss of plasma membrane integrity, and apoptosome formation [156, 188]. Notably, necroptosis is always accompanied by significant inflammation and an immune response [188]. Necroptosis is initiated by ligation between death receptors (particularly TNF-R1) and RIPK1, which acts upstream to phosphorylate and activate RIPK3. Activation of RIPK3 is required for the recruitment and phosphorylation of Mixed Lineage Kinase Domain-Like (MLKL) protein. Oligomerized MLKL acquires the ability to target the plasma membrane and cause membrane permeabilization, which is critical for the execution of necrotic cell death [189]. The core molecular complex, consisting of RIPK1, RIPK3, and MLKL, is recognized as the necrosome, a specific component in the extrinsic necrosis pathway [190]. It was proposed that mitochondrial phosphatase 5 (PGAM5) is involved in the intrinsic necrosis pathway (Figure 3(c)), since knocking it down attenuates the necrosis induced by ROS or a calcium ionophore. The necrosome can bind sequentially to PGAM5L and PGAM5S, two splicing isoforms of PGAM5. PGAM5L is a substrate for Keap-1, which acts as a thiol-reactive sensor for ROS and might be related to ROS-triggered necrosis. PGAM5S, located in a relatively hydrophobic environment of mitochondria, can directly recruit and activate Drp1 on mitochondria and cause mitochondrial fragmentation, which is recognized as a common step in both apoptosis and programmed necrosis [191]. In addition, in some cases of apoptosis, the opening of the mPTP also contributes to mitochondrial swelling and rupture, as well as to necrotic cell death, which is regulated by Bax/Bak and cyclophilin D (CypD) [192, 193]. AIF is a flavin-binding protein embedded into the inner mitochondrial membrane, which renders it capable of absorbing blue light [194]. It has been implied that AIF is indispensable for cell life due to its vital properties in the assembly and stability of the mitochondrial respiratory chain, maintenance of mitochondrial morphology, regulation of oxidative stress, and cell death [195, 196]. Mitochondrial AIF release is a critical step in AIF-mediated necroptosis, which requires the sequential activation of poly(ADP-ribose) polymerase-1 (PARP-1), calpains, and Bax [197]. Calpains cleave AIF into tAIF, a soluble proapoptotic product weighing approximately 57 kDa, and Bax contributes to mitochondrial permeabilization, which facilitates the release of tAIF into the cytoplasm [198]. Cytosolic tAIF is rapidly translocated to the nucleus with the assistance of cyclophilin A (CypA), which leads to chromatinolysis and the simultaneous regulation of *γ*H2AX and CypA [199, 200] (Figure 3(d)).

Some studies have shown that mitochondria play a role in blue light-induced retinal cell necroptosis. Jaadane et al. established a model in vivo by exposing Wistar rats to constant light from residentially available white LEDs, and they found enhanced oxidative stress and a proinflammatory cytokine response in RPE cells. Moreover, cell death by programmed necrosis after extended blue light exposure was confirmed by the staining of necroptosis markers, observation of the nuclear morphology, and lack of apoptosis in their model [201]. It has also been reported that blue light-induced RGC-5 death occurs by necroptosis because RGC-5 death was found to be caspase-independent and could be suppressed by necrostatin-1, which blocks the serine/threonine kinase activity of the receptor that interacts with RIPK1 [202]. A study conducted by del Olmo-Aguado et al. further revealed that blue light causes RGC death by necroptosis through an action in mitochondria [203]. Blue light was likely to trigger both RIPK- and AIF-mediated necroptosis of RGCs in their study since the inhibition of either RIPK1/RIPK3 or AIF provided a protective effect on the blue light hazard. They found increased RIPK1 and RIPK3 mRNA and protein levels as well as concomitant activation of AIF and HO-1 in RGCs exposed to blue light illumination (465–475 nm, 250 lx), which were suppressed by necrostatin-1, except for AIF. Furthermore, downregulating RIPK1/RIPK3 had no effect on AIF, whereas silencing of AIF mRNA counteracted the blue light hazard to some extent. Hence, they argued that blue light-activated AIF is not directly associated with the RIPK1/RIPK3 pathway [203]. Nevertheless, their findings seem at odds with the finding that abolishing RIPK1 genes does not affect the mitochondrial alteration characteristic of necroptosis, such as AIF release, which means the deletion of RIPK1 eliminates the possibility of AIF-mediated necroptosis [204]. Therefore, further studies should be conducted to generate a deeper understanding of the relationship between RIPK and AIF in blue light-induced retinal cell necroptosis.

## 6. Future Directions: The Roles of Mitochondrial Dynamics in the Blue Light Hazard

It is generally believed that mitochondria are highly dynamic organelles that divide and fuse frequently to maintain balanced mitochondrial dynamics [205, 206]. Nevertheless, excessive fission occurs under stress [206]. In mammalian cells, mitochondrial fission is primarily mediated by dynamin-related protein 1 (Drp1) and its receptor, mitochondrial fission protein 1 (Fis1). Activated Drp1 could be recruited from the cytosol to the outer mitochondrial membrane to form oligomeric Drp1 complexes that wrap around and constrict the mitochondrial tubules, causing mitochondria to be divided into two halves by the use of the energy generated from the hydrolysis of GTP [207, 208]. Fis1 is one of the mitochondrial outer membrane proteins and is involved in the fragmentation and perinuclear aggregation of mitochondria [209]. Mitochondrial fragmentation is associated with Bax activation, outer membrane permeabilization, increased intracellular ROS levels, and ATP depletion, which ultimately leads to cell death [210, 211]. Fortunately, mitochondrial fission is balanced by physiological fusion. The mitochondrial fusion proteins mitofusin 1 (Mfn1) and Mfn2 and optic atrophy 1 (OPA1) mediate mitochondrial outer membrane and intimal fusion, respectively [206, 212, 213].

There is a relatively small body of literature that is concerned with mitochondrial morphology alterations after blue light exposure. Roehlecke et al. [179] performed a study on ARPE-19 cells in vitro and found that, after 72 h of blue light irradiation, the mitochondrial morphology changed significantly. They observed a small number of mitochondria with either small or abnormally elongated profiles. Knels et al. [214] compared mitochondrial morphology changes between two different LED arrays with peak wavelengths between 411 nm and 470 nm, respectively. They discovered that mitochondria in R28 cells exposed to blue light at 411 nm appeared collapsed or fragmented, while no morphological changes were observed at 470 nm. Marie et al. [126] carried out a series of experiments on primary RPE cells exposed to blue light and found perinuclear clustering of mitochondria with a decrease in MMP. These mitochondrial morphological changes induced by blue light are similar to those observed during mitochondrial fission and are related to mitochondrial dynamics. A recent study performed by Li et al. [182] also revealed that RGC mitochondria were changed from tubular, thread-like networks into fragmented, punctate dot-like shapes after blue light irradiation. They found that Drp1 was significantly upregulated, while the expression of Mfn2 was significantly decreased. Interestingly, the Drp1 inhibitor Mdivi-1 and Drp1 RNAi not only attenuated the changes described above in RGCs but also alleviated the ROS production, MMP decrease, and apoptosis induced by blue light.

Therefore, the disruption of mitochondrial dynamics may cause severe impairment in mitochondrial structure and functions and is thus responsible for the blue light hazard to the retina and may be a novel direction for future research.

## 7. Conclusions

Because of the rapid development of LED technology, the blue light hazard not only has attracted consumers' attention but also has encouraged the study of the blue light hazard to the retina. The mitochondrion is a multifunctional organelle that plays essential roles in bioenergetics, biosynthesis, and signaling [215]. Therefore, the optimal mitochondrial functions are of vital importance in the maintenance of cell viability. Mitochondria are abundant in retinal tissues, which have more access to blue light, and the mitochondrial respiratory chain contains chromophores that absorb blue light. As a consequence, excessive blue light exposure breaks the delicate balance between the oxidant and antioxidant systems within mitochondria via photochemical effects, leading to ROS accumulation and oxidative stress. Blue light-induced photochemical damage can morphologically and functionally affect retinal mitochondria and trigger mitochondria-involved death signaling pathways, which ultimately result in irreversible cell death. In this sense, mitochondria are likely to be potential targets and initiators of the blue light hazard to the retina; therefore, they are expected to become attractive targets for preventive and therapeutic treatments that rescue blue light-induced retinal injury. Of course, the rational use of LED devices is the first step in preventing the blue light hazard.

## Figures and Tables

**Table 1:** Features of major chromophores or pigments in the mitochondria or the retina.

Chromophores^∗^ or pigments			Wavelength absorption maxima (nm)	Molecular or cellular localizations	Roles in the blue light hazard	References
Mitochondria	Flavin	FMN	450	Complex I	Pro-^†^	[128, 216]
		FAD		Complex II		
	Porphyrin	Hemes	400-410	Complex III		[135, 217, 218]
				Cytochrome c oxidase (complex IV)		
				Cytochrome P450		
Retina	Blue-cone opsin^‡^ (OPN1SW)		430	Outer segments of cones		[79, 219]
	Rhodopsin (OPN2)		500	Outer segments of rods		[220, 221]
	Melanopsin (OPN4)		479	Cell membrane of ipRGCs		[222, 223]
	All-*trans*-retinal		382	POS; phagocytosed POS in RPE		[78, 224]
	A_2_E^§^		336, 430-439	Lipofuscin in RPE cells		[225, 226]
	Melanin		335	Melanosomes in RPE cells	Anti-/pro-	[86, 98, 227]
	Carotenoid	Lutein	450	HL and OPL in the peripheral retina	Anti-^†^	[228–231]
		Zeaxanthin		HL and OPL in the macula		

Abbreviations: FMN: flavin mononucleotide; FAD: flavin adenine dinucleotide; complex I: NADH dehydrogenase; complex II: succinate dehydrogenase (SDH); complex III: coenzyme Q – cytochrome c reductase; OPN: opsin; POS: photoreceptor outer segments; HL: Henle's nerve fiber layer, composed of photoreceptor axons; OPL: outer plexiform layer. ^∗^The (part of the) molecule that absorbs the radiation and accounts for its color is dubbed the chromophore. ^†^Pro- refers to the corresponding chromophore or pigment that can mediate the blue light hazard, while anti- refers to a protective effect against the blue light hazard. ^‡^Cone opsins include three visual pigments, which are sensitive to blue light (peak at 430 nm), green light (peak at 540 nm), and red light (peak at 570 nm), respectively. ^§^A2E is one of the components and the potent photosensitizers of lipofuscin. Absorption spectrum of A2E is featured by two maxima at 336 and 430–439 nm.
